# Cellular Fibronectin Containing Extra Domain A Causes Insulin Resistance via Toll-like Receptor 4

**DOI:** 10.1038/s41598-020-65970-6

**Published:** 2020-06-04

**Authors:** Sangam Rajak, Yusuf Hussain, Khushboo Singh, Swasti Tiwari, Basir Ahmad, Sachi Bharti, Prem Prakash

**Affiliations:** 1 Department of Molecular Medicine & Biotechnology, Sanjay Gandhi Postgraduate Institute of Medical Sciences, Lucknow, Uttar Pradesh 226014 India; 2 Institute of Molecular Medicine - Jamia Hamdard, Hamdard Nagar, New Delhi, Delhi 110062 India; 3 Department of Pharmacology, CSIR-Central Drug Research Institute, Lucknow, 226031 India

**Keywords:** Biomarkers, Diagnostic markers, Predictive markers, Prognostic markers, Disease prevention, Lifestyle modification, Nutritional supplements, Preventive medicine

## Abstract

We determined the role of cellular fibronectin (CFN) containing the alternatively spliced extra domain A (FN-EDA) in causing insulin resistance (IR) through toll-like receptor 4 (TLR4). Circulating FN-EDA level was evaluated in mouse and rat IR models. Specific anti-FN-EDA antibody and TLR4 inhibitor were used to study its role in IR in mice. CFN protein was injected to evaluate TLR4 dependent effect of FN-EDA in IR. Furthermore, FN-EDA was estimated in blood plasma and correlated with demographic and clinical characteristics in healthy human participants (n = 38). High-fat diet feeding significantly increased circulating FN-EDA in both mouse (P = 0.03) and rat (P = 0.02) IR models. Antibody against FN-EDA protected mice from IR by increasing glucose disposal rate following glucose (P = 0.02) and insulin (P = 0.01) tolerance tests. CFN protein injection caused IR, however, TLR4 inhibitor protected the mice from CFN induced IR. Multivariate regression analysis predicted an independent positive correlation between circulating FN-EDA and fasting plasma glucose (P = 0.003) in healthy human participants. In conclusion, FN-EDA may cause IR through TLR4 by decreasing glucose disposal rate following glucose and insulin load. Targeting FN-EDA thus can be considered as a possible therapeutic strategy to delay prediabetes progression to diabetes.

## Introduction

Diabetes is an impaired metabolic condition that causes a major health problem worldwide^1–3^. Prediabetes is an intermediate state between normal glucose tolerance and clinical manifestation of diabetes. Insulin resistance (IR) is the hallmark feature of prediabetes and diabetes caused by endothelial dysfunction and low-grade inflammation^4–7^. Defective endothelium releases cellular fibronectin (CFN) into the blood plasma which found to be very low in healthy human and get elevated in diabetes. Diabetes patients were tested with a high level of CFN compared to ischemic stroke and renovascular hypertensive patients^8^. Supervised aerobic training significantly reduces circulating CFN in diabetes patients. Circulating CFN level in diabetes patients was significantly correlated with fasting plasma glucose (FPG), glycated haemoglobin, fasting insulin and IR. Aerobic exercise improved endothelial dysfunction and thus reduced circulating CFN level in diabetes subjects^9^. Both studies suggested circulating CFN level may be considered as a biomarker for endothelial cell activation in diabetes. However, the role of CFN as a damage-associated molecular pattern (DAMP) has not been tested in causing diabetes.

CFN has multiple isoforms generated by alternative processing of a single primary transcript at three sites extra domain A (EDA), extra domain B (EDB), and the type III homologies connecting segment (IIICS)^10,11^. Notably, cellular FN-EDA is present in the endothelium of atherosclerotic, but not healthy arteries^12,13^. Alternative splicing of the EDA exon, but not EDB, is specifically regulated in several physiological and pathological processes, including lung, liver and kidney fibrosis^14–16^, cutaneous wound healing^17,18^, lymphatic valve morphogenesis^19^, vascular intimal proliferation^13^, vascular hypertension^20^, and cardiac transplantation^21^, suggesting a functional role for the FN-EDA in these processes.

FN-EDA is an endogenous ligand for Toll-like receptor 4 (TLR4)^22–24^. We have shown FN-EDA activates platelet Toll-like receptor 4 (TLR4) and thus accelerated ferric chloride induces carotid artery thrombosis in mice^24^. FN-EDA aggravates ischemic stroke via TLR4 dependent activation of thrombo-inflammation in a model of hypercholesterolemia, the apolipoprotein E-deficient (Apoe^−/−^) mice^23^. Moreover, FN-EDA exacerbates atherosclerosis in Apoe^−/−^ mice fed on a high-fat Western diet for 14 weeks^25^. We showed for the first time that FN-EDA activates macrophage TLR4 in mice aortic lesions and human coronary artery atherosclerotic plaques^25^. FN-EDA aggravated myocardial reperfusion in TLR4 dependent manner in hyperlipidaemic mice by causing thrombo-inflammation^22^.

Since endothelial dysfunction and TLR4 activation causes metabolic dysfunctions^26–29^, we tested our hypothesis FN-EDA contributes to the development of IR via TLR4. We used rodent IR models to study the role of FN-EDA as a DAMP protein and to corroborate our findings evaluated circulating FN-EDA level in the healthy human subject. Our experimental evidence indicates FN-EDA may cause IR through TLR4 in mice and may help diagnose prediabetes in the human subject.

## Results

### High circulating FN-EDA in HFD fed IR rodent

**Figure 1:**
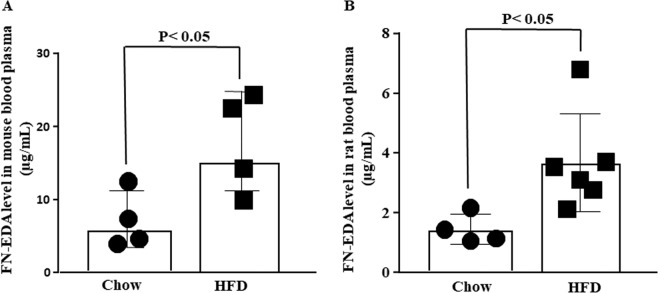
High circulating FN-EDA in HFD fed IR rodent. (**A**) Bar diagram depicts circulating FN-EDA level in male mice blood plasma. FN-EDA level in mice blood samples was estimated following HFD feeding for ten-weeks. Antibody specific for FN-EDA was used for the estimation by ELISA. HFD feeding for ten-weeks causes a significant increase in circulating FN-EDA level in male mice comparable to chow-fed mice. (**B**) Bar diagram represents circulating FN-EDA in rats blood plasma. Female rats were fed on HFD for one-week following STZ and NA infusion. Circulating FN-EDA was significantly increased following HFD feeding in rat compared to chow-fed control. Data represented as mean (SD) and P < 0.05 considered statistically significant.

To test our hypothesis whether FN-EDA modulates IR we first tested the circulating level FN-EDA in two rodent models of IR. Circulating level of FN-EDA was found to be very low in healthy human however significantly get increased in diabetes^8,30^. If FN-EDA modulates IR its plasma concentration should correlate with metabolic diseases condition. We fed C57BL/6 male mice on HFD for ten weeks and Wistar female rats were kept on HFD for 4 days after subjected to NA and STZ infusion. A specific antibody was used to determine the circulating level of FN-EDA by ELISA. Significant increase in body weight over time following HFD feeding was observed in mice (Supplementary Fig. 1A,B) however in rat found comparable to chow-fed control (Supplementary Fig. 2A). FPG was significantly increased in HFD fed mice (Supplementary Fig. 1C) and rats (Supplementary Fig. 2B). Significantly high level of plasma FN-EDA (Fig. 1A,B) compared to control were noted in both rodent models of IR. IPGTT and IPITT were significantly impaired in both mice (Supplementary Fig. 1D,E and F,G respectively) and rats (Supplementary Fig. 2C,D and 2E-F).

### High circulating FN-EDA in one-week HFD fed IR mice

**Figure 2:**
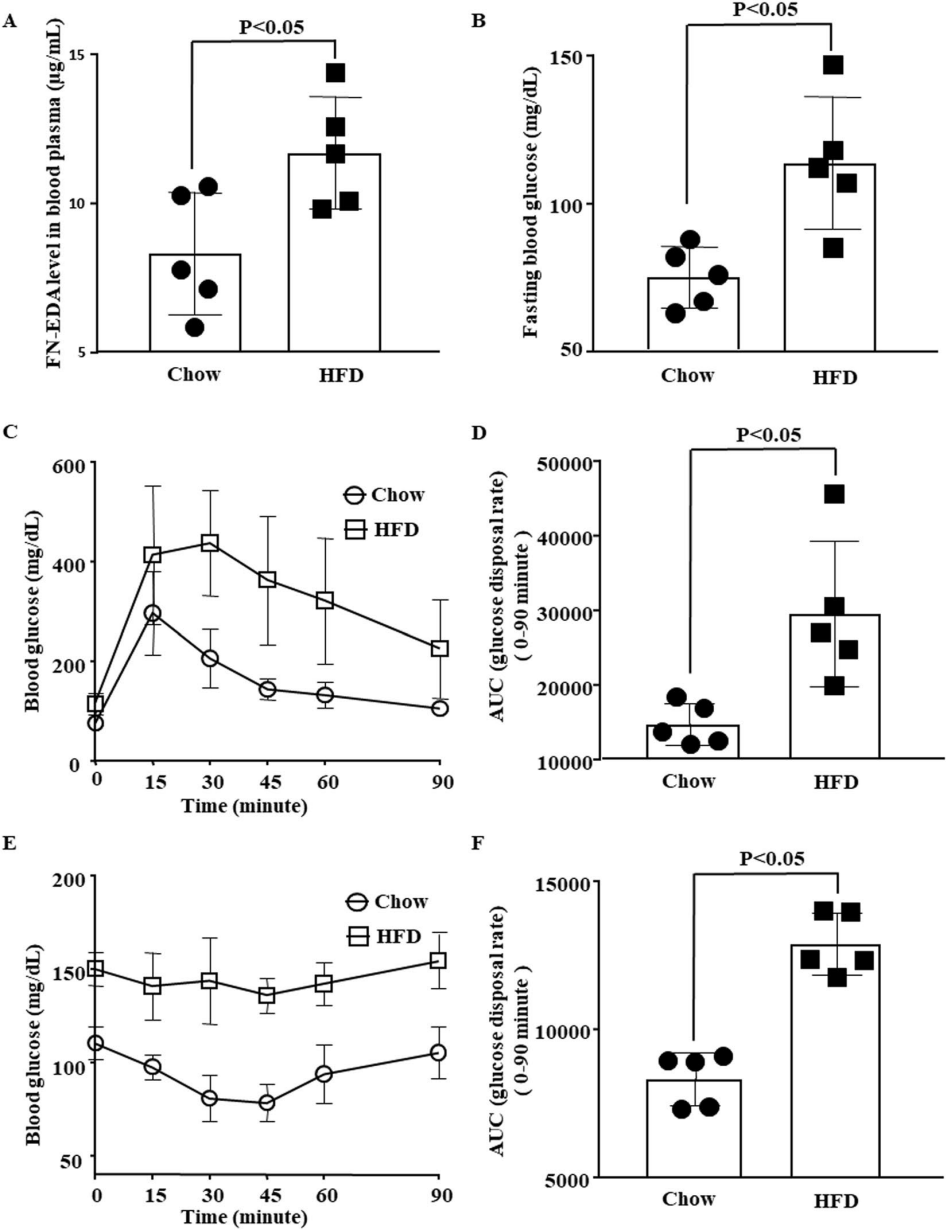
High circulating FN-EDA in one-week HFD fed IR mice. (**A**) Bar diagram represents the circulating level of FN-EDA in male mice blood plasma following HFD feeding for one-week. HFD feeding for one-week significant increase FN-EDA in male mice. (**B**) Bar diagram depicts FPG in one-week HFD fed male mice. Glucometer was used to measure blood glucose in 6 hours fasted male mice. HFD feeding for one-week significant increases FPG in male mice compared to chow-fed mice. Line and bar graph represent glucose disposal following (**C** and **D**) IPGTT and (**E** and **F**) IPITT in male mice kept on HFD for one-week. Blood samples were collected from the tail vein at various time points starting from time zero to until 90 minutes following glucose and insulin infusion. Glucose disposal was significantly impaired overtime in HFD fed mice following glucose and insulin tolerance tests. Data represented as mean (SD) and P < 0.05 considered statistically significant.

We noted a significantly high level of FN-EDA in mice fed on HFD for ten-weeks. Experimental interventions to modulate cell signalling pathways in long term HFD fed mice often get challenging, costly and time-consuming. Therefore, we opted for well established short term HFD induced IR model in mice^31,32^. C57BL/6 male and female mice were maintained on HFD diet for one-week followed by IPGTT and IPITT. FN-EDA level was significantly increased in mice on HFD for one-week (Fig. 2A). Significant increase in FPG level was observed compared to mice on a chow diet (Fig. 2B). However, bodyweight was found comparable in HFD and chow-fed groups (data not shown). Mice on HFD for one-week shown significantly impaired glucose tolerance as depicted by delayed glucose disposal rates in response to glucose load compared to mice on a chow diet (Fig. 2C,D). Insulin sensitivity was significantly impaired as represented by decreased basal glucose disposal rates following insulin load compared to chow control (Fig. 2E,F). Metabolic dysfunction and circulating FN-EDA levels observed in male mice (Fig. 2) on HFD for one-week was independent of gender (Supplementary Fig. 3).

### Antibody against FN-EDA protects from IR

**Figure 3:**
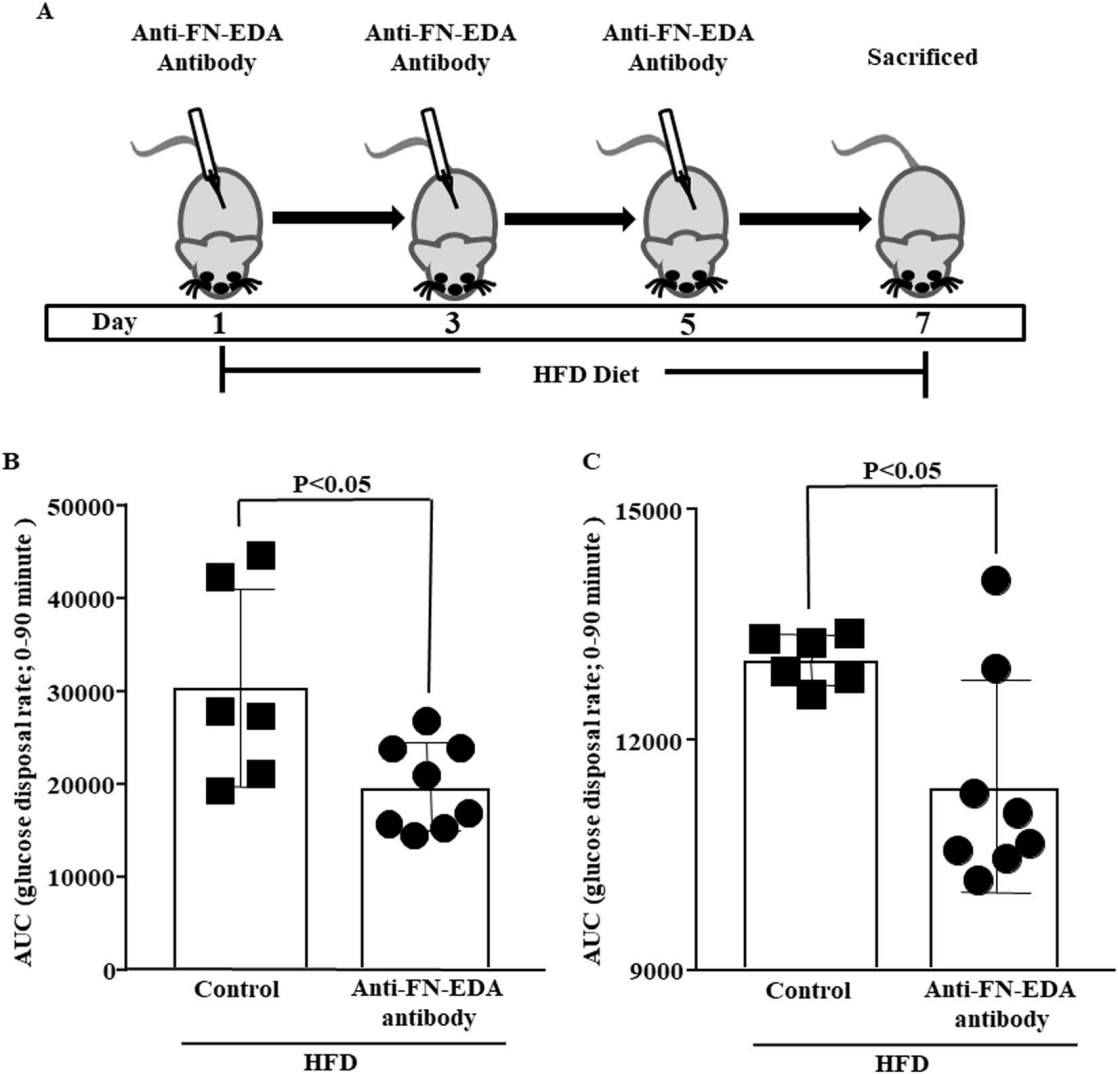
Antibody against FN-EDA protects from IR. (**A**) Mice on HFD for one-week were treated with a monoclonal antibody specific to FN-EDA three times at 48 hours interval. The first dose was administered just before keeping the mice on HFD. The bar graph depicts the effect of FN-EDA inhibition on glucose disposal following (**B**) IPGTT and (**C**) IPITT. Monoclonal anti-fibronectin antibody clone 3E2 specific to FN-EDA was infused to mice on HFD for one-week. Antibody against FN-EDA significantly increased glucose deposal rate following glucose and insulin load. Data represented as mean (SD) and P < 0.05 considered statistically significant.

To determine FN-EDA mediates IR, we infused monoclonal anti-fibronectin cellular antibody clone FN-3E2 specific for FN-EDA to HFD fed mice. Our previous published studies suggest control-Ig isotype does not significantly changes circulating FN levels and mice were normal during and after treatment^22,23^. Thus, we used normal saline as vehicle control. Antibody and saline treatment did not significantly change FN level (data not shown) and mice were normal on or after the treatment. Anti-FN-EDA antibody treatment ameliorated impaired glucose tolerance by significantly increasing glucose disposal rate in response to glucose load compared to normal saline-treated mice fed on an HFD for one-week (Fig. 3B). Insulin sensitivity was also ameliorated by significantly increasing basal glucose disposal rate following insulin load compared to saline-infused mice fed on the HFD for one-week (Fig. 3C).

### TLR4 inhibitor protects from IR

We tested the protective effect of TLR4 inhibitor against HFD induced IR to justify its FN-EDA mediated association in causing IR. A specific TLR4 inhibitor TAK-242 was given to mice fed on the HFD. TAK-242 treatment protected mice from HFD induced IR. TAK-242 significantly increased glucose disposal rate following glucose (Supplementary Fig. 4B) and insulin (Supplementary Fig. 4C) load in one-week HFD fed mice compared to vehicle-treated control.

### CFN causes IR through TLR4

**Figure 4:**
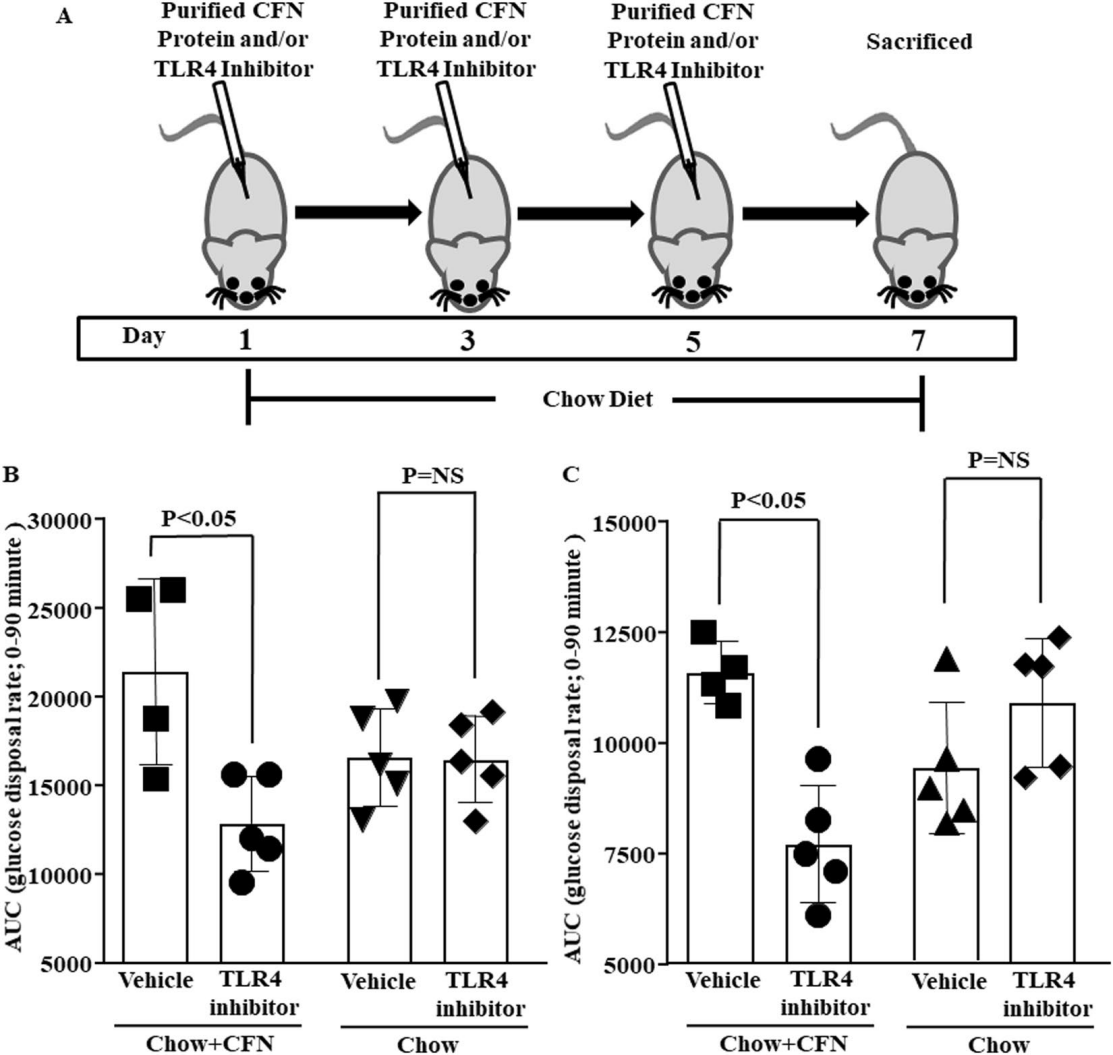
CFN causes IR through TLR4. (**A**) Mice on a chow diet were infused with purified CFN protein and TLR4 inhibitor three times at 48 hours interval. Bar graphs depict area under the curve (AUC) of glucose utilization rate following (**B**) IPGTT and (**C**) IPITT. Chow fed mice were injected with purified CFN protein along with TLR4 inhibitor. TAK-242 ameliorated purified CFN protein-mediated IR by significantly decreasing AUC of glucose utilization rate following IPGTT and IPITT. However, mice injected with TAK-242 alone shown AUC comparable to vehicle-treated mice. Data represented as mean (SD) and P < 0.05 considered statistically significant.

To investigate the specific role of TLR4 dependent FN-EDA in causing IR, mice on the chow-fed diet were subjected to purified CFN protein and/or TAK-242. CFN infusion renders mice to IR fed on a chow diet. CFN caused a significant decrease in glucose disposal rate following glucose (Fig. 4B) and insulin (Fig. 4C) load. TLR4 signalling suppressor protected the mice against CFN induced IR. TAK-242 given in combination with CFN significantly decreases area under the curve (AUC) of glucose disposal following glucose (Fig. 4B) and insulin (Fig. 4C) load. However, treatment with only TLR4 inhibitor shows glucose disposal AUC comparable to vehicle-treated control (Fig. 4B and C).

### High circulating FN-EDA in prediabetes human subjects

**Figure 5:**
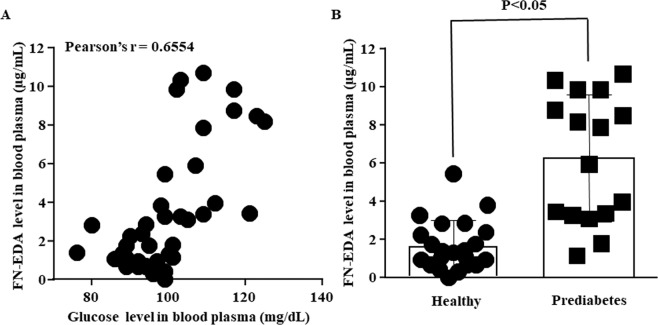
High circulating FN-EDA in prediabetes human subjects. (**A**) Scatter plot depicts the correlation between FN-EDA and FPG in healthy human subjects. Healthy human subjects were tested for FN-EDA and FPG. Circulating FN-EDA level significantly positively correlated with FPG level in healthy subjects. (**B**) Bar diagram depicts circulating FN-EDA level in disease conditions. Healthy subjects were divided into two groups based on FPG. Healthy human subjects having FPG ≤ 100 mg/dL and prediabetes patients have ≥100 to 125 mg/dL. Circulating FN-EDA level was significantly increased in prediabetes patients compared to the healthy human subjects. Data represented as mean (SD) and P < 0.05 considered statistically significant. (healthy subject n = 22, prediabetes n = 16).

FN-EDA found to be very low in healthy subject and its level gets significantly increased in diabetes^8,30^. Thus FN-EDA may be considered as a biomarker for diabetes. However, to implicate FN-EDA as a DAMP, its circulating level should be high in borderline diabetes state such as prediabetes. Healthy human volunteers (n = 38) were recruited to investigate the circulating level of FN-EDA and its correlation with FPG and other independent variables. Univariant regression analysis shows age, waist size and FPG were significantly associated with plasma levels of FN-EDA (Table 1). Each unit increase in age, waist size and FPG causes 0.157 µg/ml, 0.392 µg/ml and 0.189 µg/ml increase in plasma FN-EDA level in healthy human subject respectively. Multivariant regression model further confirmed an independent association of FPG with circulating FN-EDA (Table 2). Each unit increase in FPG causes 0.131 µg/ml increase in plasma FN-EDA. Pearson’s correlation coefficient shows a positive linear correlation between plasma FN-EDA and FPG in healthy human (Fig. 5A). Based on FPG two study groups prediabetes and healthy were formed. The mean age of the prediabetes 39 (year; n = 16; 95% CI 32 to 46) and 69% were men. The mean age of the healthy 27 (year; n = 22; 95% CI 25 to 29) and 68% were men. Significant increase in circulating FN-EDA has observed in prediabetes subject compare to healthy control (Fig. 5B). Table 3 shows the unadjusted and the age, sex, BMI, waist size, systolic and diastolic blood pressure adjusted difference in FN-EDA between groups.

**Table 1:** Univariate regression analysis of variables for the prediction of the high circulating level of FN-EDA in the healthy subject (n = 38).

	Regression coefficient (95% CI)	Significance
Age (years)	0.157 (0.073 to 0.240)	P = 0.001
Body Mass Index (kg/m^2^)	0.269 (−0.039 to 0.578)	P = 0.085
Waist Size (inches)	0.392 (0.195 to 0.589)	P = 0.000
Systolic Blood Pressure (mm Hg)	−0.014 (−0.095 to 0.066)	P = 0.718
Diastolic Blood Pressure (mm Hg)	−0.029 (−0.141 to 0.083)	P = 0.604
Fasting Glucose (mg/dL)	0.189 (0.116 to 0.263)	P = 000

For each unit increase in the predictor variable, the regression coefficient shows a mean increase in circulating FN-EDA level in blood plasma. Data are regression coefficients (95% CI).

**Table 2:** Multivariant regression analysis of variables for the prediction of a high circulating level of FN-EDA in the healthy subject (n = 38).

	Regression coefficient (95% CI)	Significance
Age (years)	0.060 (−0.048 to 0.167)	P = 0.269
Body Mass Index (kg/m^2^)	−0.290 (−0.649 to 0.070)	P = 0.110
Waist Size (inches)	0.283 (−0.050 to 0.615)	P = 0.094
Fasting Plasma Glucose (mg/dL)	0.131 (0.048 to 0.213)	P = 0.003

For each unit increase in the predictor variable, the regression coefficient shows a mean increase in circulating FN-EDA level in blood plasma. Data are regression coefficients (95% CI).

**Table 3:** Unadjusted and age, sex, waist size, BMI, systolic and diastolic blood pressure-adjusted differences in plasma levels of FN-EDA in prediabetes and healthy groups.

Groups	Unadjusted difference (95% CI)	Sig.	Adjusted difference (95% CI)	Sig.
Prediabetes - Healthy	4.564 (2.979 to 6.148)	P = 000	3.750 (2.134 to 5.366)	P = 000

## Discussion

In this report, we tested the hypothesis that FN-EDA may be considered as DAMP protein possibly causes IR through its endogenous ligand TLR4. Here we have shown the various line of experimental evidence to support our hypothesis. Circulating FN-EDA was found to be significantly high in IR mice and rats fed on HFD. Specific targeting of FN-EDA by monoclonal antibody and inhibition of TLR4 protect mice from HFD induced glucose and insulin intolerance. On the other hand infusion of CFN causes delayed glucose disposal rate following glucose and insulin tolerance test. However, inhibition of TLR4 protected the mice against CFN protein-mediated impaired glucose disposal rate in chow-fed mice. Furthermore, the multivariate regression model predicted a positive correlation between FN-EDA and FPG in the healthy human participant.

Circulating FN-EDA level in diabetes subjects were found to be significantly high compared to renovascular hypertensive and healthy control subjects^8^. Diabetes patients associated with cardiovascular risk factors were having high circulating FN-EDA compared to those devoid of such risk factors^30^. Elevated triglyceride, current or past cigarette smoking, and higher urinary albumin excretion were found to be independently associated with plasma FN-EDA in diabetes patients. Diabetes patients underwent supervised aerobic exercise for 12 weeks showed a significant reduction in plasma FN-EDA and IR^9^. Our results are in corroboration with the previous finding show a significant increase in plasma FN-EDA level in prediabetes human subjects. We found age, waist size and FPG positively correlates with plasma FN-EDA level in healthy subjects. Moreover, circulating FN-EDA independently associated with FPG. IR is a hallmark of prediabetes and diabetes and an independent risk factor for cardiovascular diseases (CVD). High level of circulating FN-EDA in mice and rats IR models further confirmed our findings in the human subject. Lowering plasma FN-EDA may help ameliorate IR as previously reported study observed decreasing plasma FN-EDA significantly ameliorated IR in diabetes subjects underwent specialized aerobic exercise. Inhibition of FN-EDA by specific monoclonal antibody protected mice from HFD induced glucose and insulin intolerance. We further confirm our observation by infusing CFN protein to develop IR in chow-fed mice. TLR4 is well documented as an endogenous ligand for FN-EDA^22–24,33,34^, thus we inhibited TLR4 to reverse the effect of CFN protein in chow-fed mice. Inhibition of TLR4 protected the mice against CFN induced IR, however, showed a comparable effect when given to control mice.

Diabetes once diagnosed can not be cured and patients need to be on medication for a lifetime. However, borderline diabetes or prediabetes can be cured by adopting a healthy living lifestyle. Thus it is important to capture diabetes at its early stages of development to help patients to live a disease-free life. Published reports proposed FN-EDA as a biomarker for diabetes and its associated CVD^8,9,30^. However, in the present study, we are proposing FN-EDA as a DAMP protein may cause IR in mice. Endothelial activation is well documented in IR however a direct regulating role of extracellular matrix protein in causing IR is not known. Our published report confirmed endothelial dysfunction might release extracellular matrix protein such as FN-EDA. FN-EDA may activate hematopoietic TLR4 most probably platelet TLR4 and thus causes a prothrombotic and proinflammatory state^22–24^. Thrombotic complications, low-grade inflammation and TLR4 activation are well reported in IR and thus we tested the role of FN-EDA in causing IR. We found a significant increase in circulating FN-EDA in prediabetes human subject and positively correlates with age, waist size and FPG. FPG independently associated with prediabetes, every unit increase in FPG increases 0.13 µg/ml (0.05 to 0.21, 95% CI) circulating FN-EDA in a healthy human subject. Our data predict after age 32 healthy people in India may tend to exercise less which may cause an increase in waist size and FPG. Thus, plasma FN-EDA level may be monitor after age 32 in Indian healthy population to capture diabetes at its early stages of development. Healthy lifestyle changes may decrease its plasma concentration and protect from prediabetes and its progression to diabetes. Furthermore, our antibody-mediated FN-EDA inhibition and CFN injection results confirmed a regulatory role of FN-EDA in IR. FN-EDA may be considered as a therapeutic target for the treatment of diabetes at its early stages of development. FN-EDA is distinct from plasma fibronectin having alternatively spliced EDA domain thus may be considered as a novel therapeutic target in case of prediabetes and diabetes with less unwanted side-effects. We found high plasma FN-EDA either through HFD feeding or CFN infusion activates TLR4 and thus causes IR in mice. Role of TLR4 in causing IR is well documented^27,35–37^ however its regulation by FN-EDA in IR is not known.

It is well appreciated that chronic low-grade inflammation associated with prolonged overnutrition may cause IR. Similarly, we also found 10-weeks of HFD feeding developed IR in mice and significantly increases tumour necrosis factor-alpha (TNF-α) and interleukin 6 (IL-6) in blood plasma (data not shown). However, one-week HFD feeding did not alter circulatory pro-inflammatory cytokines (data not shown)) but causes IR in mice. Our findings are in corroboration with the published reports suggests one-week HFD feeding can cause IR^31,32^, however, need long term feeding to induce tissue inflammation in mice^31,38^. Short-term HFD feeding can cause an acute inflammatory response and may impair insulin signalling. One day of HFD feeding is sufficient to induce whitening and IR in brown adipose tissue (BAT) in mice. Three-days of HFD feeding reduced mitochondrial oxidation and caused macrophage infiltration in BAT^39^. Adipocytes TLR4 knockout mice found significantly more glucose tolerant when subjected to hyperinsulinemic-euglycemic clamp fed on HFD for three-weeks. Short-term HFD feeding did not cause significant changes in subcutaneous white adipose tissue and mice did not gain much weight. Even showed improved whole-body insulin sensitivity following lard oil infusion during the clamp fed on chow diet^40^. It has been suggested that a complex interconnected whole-body network exists for glucose disposal in both chronic and acute low-grade inflammation^40,41^. Reports indicate that HFD or TLR4-ligand may cause metabolic dysfunction by altering myelopoiesis via activation of TLR4 signalling pathways in hematopoietic stem cells (HSCs) through an inflammation-independent mechanism^41,42^. Hematopoietic stem and progenitor cells (HSPCs) patrol peripheral tissue through blood and lymph before returning to bone marrow (BM). These migratory HSPCs induces BM myeloid-biased following an encounter with TLR4 ligand^43^. FN-EDA is an endogenous ligand for TLR4^24,34,43,44^, our data indicates FN-EDA possibly induces BM myeloid-biased which may lead to IR in mice.

Modulatory role of FN-EDA in IR is not known, our experimental findings demonstrate that FN-EDA may cause IR through its endogenous ligand TLR4. We are proposing FN-EDA as a DAMP protein, may not only consider as a disease biomarker in prediabetes perhaps causes IR in mice. Plasma FN-EDA level may help identify prediabetes patients in a healthy human study population along with FPG. We confirmed our finding in two well-established mice and rats models of IR. We tested our hypothesis at both chronic and short term HFD feeding and in both male and female mice IR models. Modulated plasma FN-EDA level by specific antibody and purified protein and proposed a potential mechanism by inhibiting its endogenous ligand TLR4. However, a prospective cohort study warranted in a large population to study the association between circulating FN-EDA and prediabetes and its progression to diabetes. Genetically engineered mice models should be used to establish a direct role of FN-EDA in causing IR. Nevertheless, the present study may indicate a novel potential role of FN-EDA in IR, the hallmark of prediabetes and diabetes.

The contribution of proposed studies is significant because identifying the mechanisms by which FN-EDA mediates IR can be translated into clinical practice for early detection and treatment of the disease. Knowledge gained by the present work is anticipated to significantly contribute to a broader understanding of how endogenous FN-EDA and its receptors can be modulated as a therapeutic approach. We had reported a high level of circulating FN-EDA in hyperlipidemic apolipoprotein E-deficient (ApoE^−/−^) mice^23^. ApoE knock-out mice were found to be more susceptible to acute myocardial reperfusion injury and stroke outcomes. However genetic deletion of FN-EDA protected the ApoE knock-out mice against such cardiovascular acute conditions^22,23^. FN-EDA binds to hematopoietic TLR4 most likely through platelet TLR4 and thus accelerates intravascular thrombosis and leads to cardiovascular complications such as myocardial reperfusion injury and stroke^24^. Diabetes patients were at high risk for developing CVD and circulating FN-EDA level was found to be high in diabetes patients^8^. Thus, FN-EDA may be considered as one of a potential causative factor in diabetes-associated CVD. The outcomes of this work may have important clinical implications for both treatment and biomarker discovery.

## Materials and Methods

### Materials

Cellular fibronectin, from human foreskin fibroblasts (cat. no. F2518), Monoclonal anti-fibronectin cellular antibody produced in mouse clone 3E2 (cat. no. F6140), Anti-Fibronectin antibody produced in rabbit (F3648), TLR4 inhibitor TAK-242 (cat. no. 614316), Nunc-Immuno MicroWell 96 well solid plates (cat. no. M9410) were procured from Sigma-Aldrich, MO, USA). Fetal bovine serum and D-Glucose (cat. no. 50-99-7) were procured from Gibco, Applied biosystems, Bangalore, India. Streptozotocin (STZ) extra pure (cat. no. 14653) and Nicotinamide extra pure (cat. no. 72860) were procured from Sisco Research Laboratories Pvt. Ltd. Delhi, India. TMB Substrate Solution (cat. no. N301) from Thermo Scientific Waltham, MA, USA). Goat anti-rabbit IgG H&L (cat. no. ab6721) and goat anti-mouse IgG H&L (cat. no. ab6789) from Abcam, MA, USA. Insulin (Huminsulin R 100 IU Injection) from Eli Lily and Company (India) Pvt. Ltd. Glucometer (On Call Plus G113-214) and blood glucose test strips (On Call Plus, G133-119) were procured from ACON Biotech (Hangzhou) Co. Ltd., Hangzhou, China.

### HFD composition and source

High fat (ingredients composition in percentage: casein 26.5, L-cystine 0.4, maltodextrin 16.0, sucrose 9.0, lard 31.0, soyabean oil 3.0, cellulose 6.55, mineral mixture AIN 93 G 4.8, calcium phosphate dibasic 0.34, vitamin mixture AIN 93 VX 2.1 and choline bitartrate 0.3) and chow diet (ingredients composition in percentage: wheat flour 22.5, roasted Bengal gram flour 60.0, skim milk powder 5.0, casein 4.0, refined groundnut oil 4.0, mineral mixture 4.0 and vitamin mixture 0.5) were procured from ICMR- National Institute of Nutrition, Hyderabad, India.

### Animals study

Wild-type C57BL/6 mice either sex was purchased from CSIR- Central Drug Research Institute, Lucknow, India. Wistar female rats 8-10 weeks old provided by Sanjay Gandhi Postgraduate Institute of Medical Sciences, Lucknow, India (SGPGIMS). All the animal experiments were performed according to the ARRIVE guidelines and carried out in accordance with the National Institutes of Health guide for the care and use of laboratory animals (NIH Publications No. 8023, revised 1978). The study was conducted after the approval from the Animal Ethical Committee (protocol number P-02/20/2017) of SGPGIMS (Registration no. 57/ PO/ ReBi/ SL/ 99/CPCEA).

### Human study

The study was performed according to the Helsinki Declaration, and the protocol was approved by the SGPGIMS human ethics committee. Clinically healthy participants (n = 38) aged 20‐60 years were recruited after written informed consent. All the healthy subjects recruited in the study fasted as defined no caloric intake for at least 8 hours. FPG was tested using glucometer and 2 ml of blood samples were collected in ethylenediaminetetraacetic acid-coated vials from 38 subjects. The collected blood samples were immediately centrifuged and plasma was isolated and stored at -80 °C for FN-EDA estimation. The participants recruited in the study had minimal physical activities until the samples were drawn. Demographic and clinical characteristics of these healthy subjects are: the mean age 32 (years; men n = 26; 95% CI 28 to 36), body mass index 24 (BMI; kg/m2; 95% CI 23 to 25), waist size 35 (inches; 95% CI 33 to 36), systolic blood pressure 127 (mm Hg; 95% CI 123 to 132), diastolic blood pressure 83 (mm Hg; 95% CI 80 to 86), FPG 100 (mg/dL; 95% CI 96 to 104). Based on FPG the human participants were divided into two study groups: Prediabetes and healthy. Diagnosis of prediabetes was based on the American Diabetes Association’s new classification standards for the prediabetes (2016)^45^. FPG 100 mg/dL (5.6 mmol/L) to 125 mg/dL (6.9 mmol/L) were kept in the prediabetes study group.

### Long term and short term HFD feeding to mice

10 to 12 weeks and 4 to 5 weeks old mice were randomly divided into two groups and kept on chow and HFD for one and ten weeks. Intraperitoneal glucose tolerance test (IPGTT, 3 g/kg) in 6 hours fasted and insulin tolerance test (IPITT, 0.25U/kg) in non-fasting condition were performed and blood plasma samples were collected for FN-EDA quantification.

### Short term HFD feeding to rat

To induce IR in rat we treated Wistar rats with a single injection of nicotinamide (NA, 170 mg/kg, IP) followed by a single injection of streptozotocin (STZ, 32.5 mg/kg, IP) after 15 min of the first injection as previously described^46^. 3 days later we placed the rat on an HFD for 4 days to induce IR. Rats were subjected for IPGTT (3 g/kg) in 6 hours fasted and IPITT (0.6U/kg) in non-fasted condition and blood plasma samples were collected for FN-EDA estimation.

### Treatment with anti-FN-EDA antibody

We infused monoclonal anti-fibronectin cellular antibody clone 3E2 (2.5 µg/g) specific for FN-EDA to one-week HFD fed mice as described^22,23^. Monoclonal anti-cellular fibronectin clone 3E2 specifically reacts with FN-EDA but not with FN-EDB, and IIICS^18,47,48^. Three dosages of antibodies were given at 48 hours interval as illustrated by the schematic in Fig. 3A. Anti-FN-EDA antibodies (45 mg/ml) stock was diluted with normal saline and infused through retroorbital plexus. The same volume of normal saline was infused to the control group. Our previous published studies suggest control Ig isotype even at 100 μg/mouse does not exert any apparent side effect and mice were normal during and after treatment^22,23^. In the present study anti-FN-EDA antibody (50 μg/20 g mouse) contains control Ig (1.8 μg/20 g mouse). Moreover, control Ig isotype treatment did not significantly affect total FN levels in the plasma^22,23^. Thus in the present study, we have used normal saline instead of control Ig isotype.

### Treatment with TLR4 inhibitor

Mice were subjected to specific TLR4 inhibitor TAK-242 (1 mg/kg) as described^33,49^. TAK-242 (5 mg/ml) stock solution was dissolved in DMSO and diluted in normal saline to make the final DMSO concentration 1%. Three dosages of TAK-242 solution (1 mg/kg) or DMSO (1%) were injected through retro-orbital plexus at 48 hours interval as illustrated by the schematic in Supplementary Fig. 4A and Fig. 4A.

### Purified CFN protein infusion

CFN (25 µg/20 g mouse) protein obtained from human foreskin fibroblast was diluted in normal saline and injected to chow-fed mice as described in Fig. 4A. Normal saline-treated mice used as control.

### Quantification of FN-EDA in plasma samples

Quantification of FN-EDA level was done by a sandwich ELISA as described^22–24^. The wells were coated overnight at 4 °C with rabbit anti-fibronectin (0.1 μg/ml) antibody. Wells were thoroughly washed 5 times with PBS containing 0.5% Tween-20. Followed by blocking with freshly prepared 10% fetal calf serum and 2% bovine serum albumin in PBS for 30 minutes at 37 °C. Subsequently, 100 µl of plasma samples diluted in blocking buffer (1:1) were incubated for one hour at 37 °C. Wells were again washed 5 times and incubated with mouse monoclonal anti-cellular fibronectin antibody clone 3E2 (0.09 mg/ml) diluted with PBS for one hour at 37 °C. Wells were washed and incubated with anti-mouse HRP conjugated secondary antibody (0.1 μg/ml) for one hour at 37 °C. After 5 washes 3, 3′, 5, 5′-tetramethylbenzidine substrate solution was added to the samples for 10 minutes. Subsequently, the colourimetric reaction was stopped with 1 N hydrochloric acid solution and samples were read in an ELISA microplate reader at A450 nm. Human purified CFN protein used as a standard for FN-EDA.

### Statistical analysis

Statistical significance was assessed by t-test for independent samples for two groups and two-way ANOVA followed by post-hoc Bonferroni test for multiple groups. Pearson’s correlation coefficient was used to measures the strength and direction of the association between FPG and circulating FN-EDA. Based on the FPG human subjects were divided into healthy and prediabetes groups. The difference between circulating FN-EDA in healthy and prediabetes groups was described by a 95% confidence interval based on the t-test for independent samples. The difference was adjusted for sex, age, BMI, waist size, systolic and diastolic blood pressure. Linear regression analysis was used to evaluate the determinant of circulating FN-EDA in healthy subjects. Sex, age, BMI, waist size, systolic and diastolic blood pressure, and FPG were used as independent variables. Independent variables having significance level ≤0.10 or less in the univariant model were further tested in the multivariant model. Data represented as mean (SD) and statistical differences were considered significant at P < 0.05.

## Supplementary information

Supplementary information.

## Data Availability

The Institutional Human Ethical Committee ethic statement and informed consent do not allow sharing individual human participant data. The animal datasets generated during and/or analyzed during the current study are available from the corresponding author upon reasonable request.
