# Supplementary material for: Cellular Fibronectin Containing Extra Domain A Causes Insulin Resistance via Toll-like Receptor 4
Source: Sci Rep. 2020 Jun 4;10:9102. doi: 10.1038/s41598-020-65970-6 (PMC7272645; doi:10.1038/s41598-020-65970-6)

Title: Cellular Fibronectin Containing Extra Domain A Causes Insulin Resistance via Toll-like Receptor 4.

Sangam Rajak[1], Yusuf Hussain[1], Khushboo Singh[1], Swasti Tiwari[1], Basir Ahmad[2], Sachi Bharti[3] and Prem Prakash[1,2, *]

[1]Department of Molecular Medicine & Biotechnology, Sanjay Gandhi Postgraduate Institute of Medical Sciences, Lucknow, Uttar Pradesh 226014.

[2] Institute of Molecular Medicine - Jamia Hamdard, Hamdard Nagar, New Delhi, Delhi 110062.

[3] Department of Pharmacology, CSIR-Central Drug Research Institute, Lucknow, India 226031.

* Corresponding author

JH-Institute of Molecular Medicine, Jamia Hamdard, New Delhi, Delhi, India-110062.

Phone # +91-11-26059688 Ext 5578, e-mail: prem@jamiahamdard.ac.in.

Supplementary figures

Supplementary figure 1.

High circulating FN-EDA in IR mouse model. Male mice were kept on chow and HFD for ten-weeks. Mice were given ad libitum access to food and water. The (A) line and (B) bar graph depict a significant increase in body weight over time following HFD feeding. Mice body weight were recorded every week until 10th week on diet. HFD significantly increases the mice body weight over time compared to chow-fed mice. (C) The bar graph shows the blood glucose level in mice fasted for 6 hours. FPG level was significantly increased in HFD fed mice. The lines and bar graphs illustrate glucose disposal rate over time following (D and E) IPGTT and (F and G) IPITT. HFD significantly impaired glucose utilization rate when mice subjected to IPGTT and IPITT. Data represented as mean (SD) and $P<0.05$ considered statistically significant.

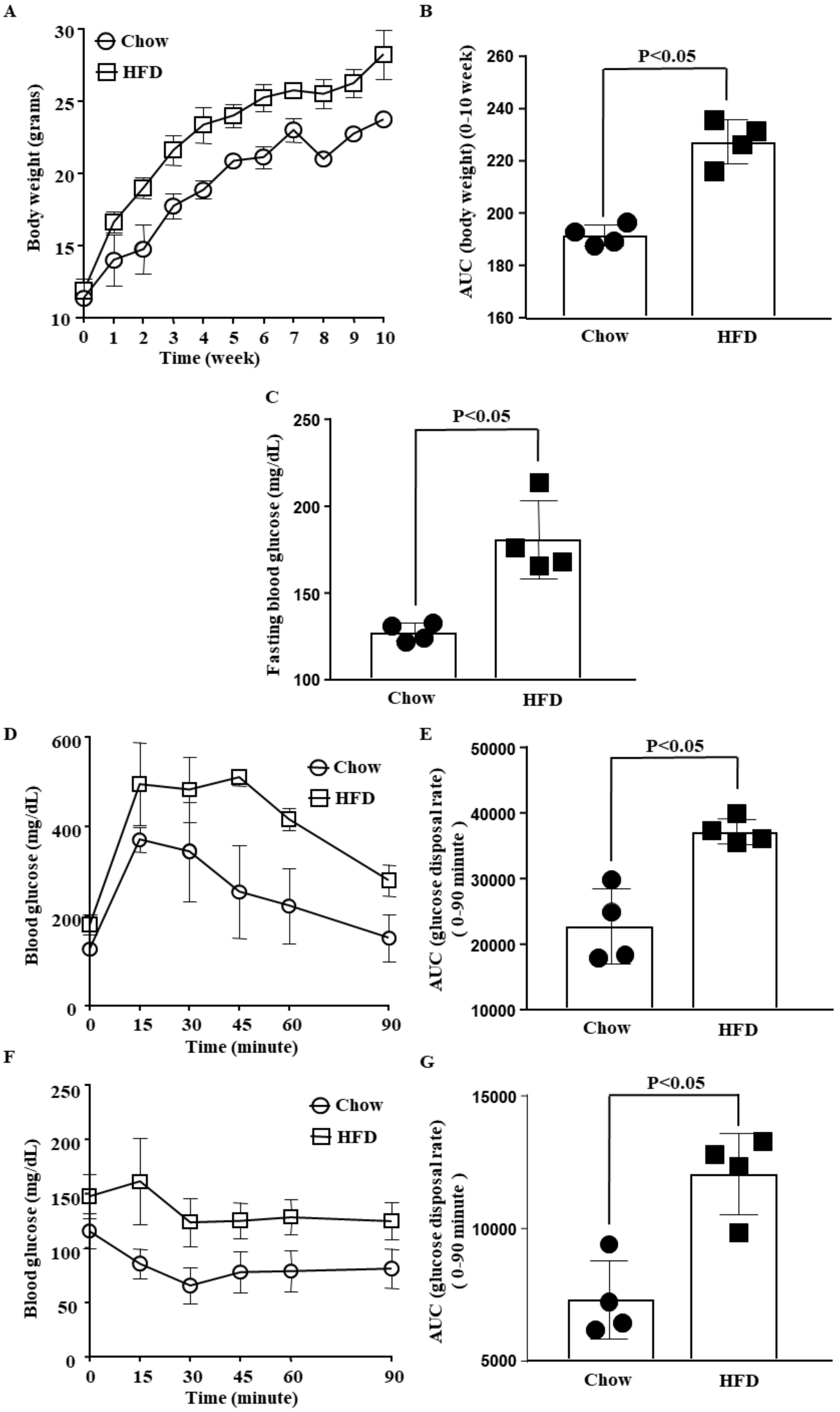
A
B
C
D
E
F
G
Chow
HFD
Body weight (grams)
Time (week)
AUC (body weight) (0-10 week)
P<0.05
Fasting blood glucose (mg/dL)
Blood glucose (mg/dL)
Time (minute)
AUC (glucose disposal rate)
(0-90 minute)

Supplementary figure 2.

IR rat model. Female rats were injected with NA (170mg/kg) and STZ (32.5mg/kg) prior to keeping on HFD for four days. The bar graphs depict (A) body weight, (B) FPG after 4 days on the HFD. HFD feeding caused a significant increase in FPG, however, bodyweight found comparable to the rats fed on chow diet. The line and bar graphs depict glucose disposal over time following (C and D) IPGTT (3g/kg) and (E and F) IPITT (0.6 IU/kg). HFD feeding causes a significant decrease in glucose disposal rate following IPGTT and IPITT. Data represented as mean (SD) and P<0.05 considered statistically significant.

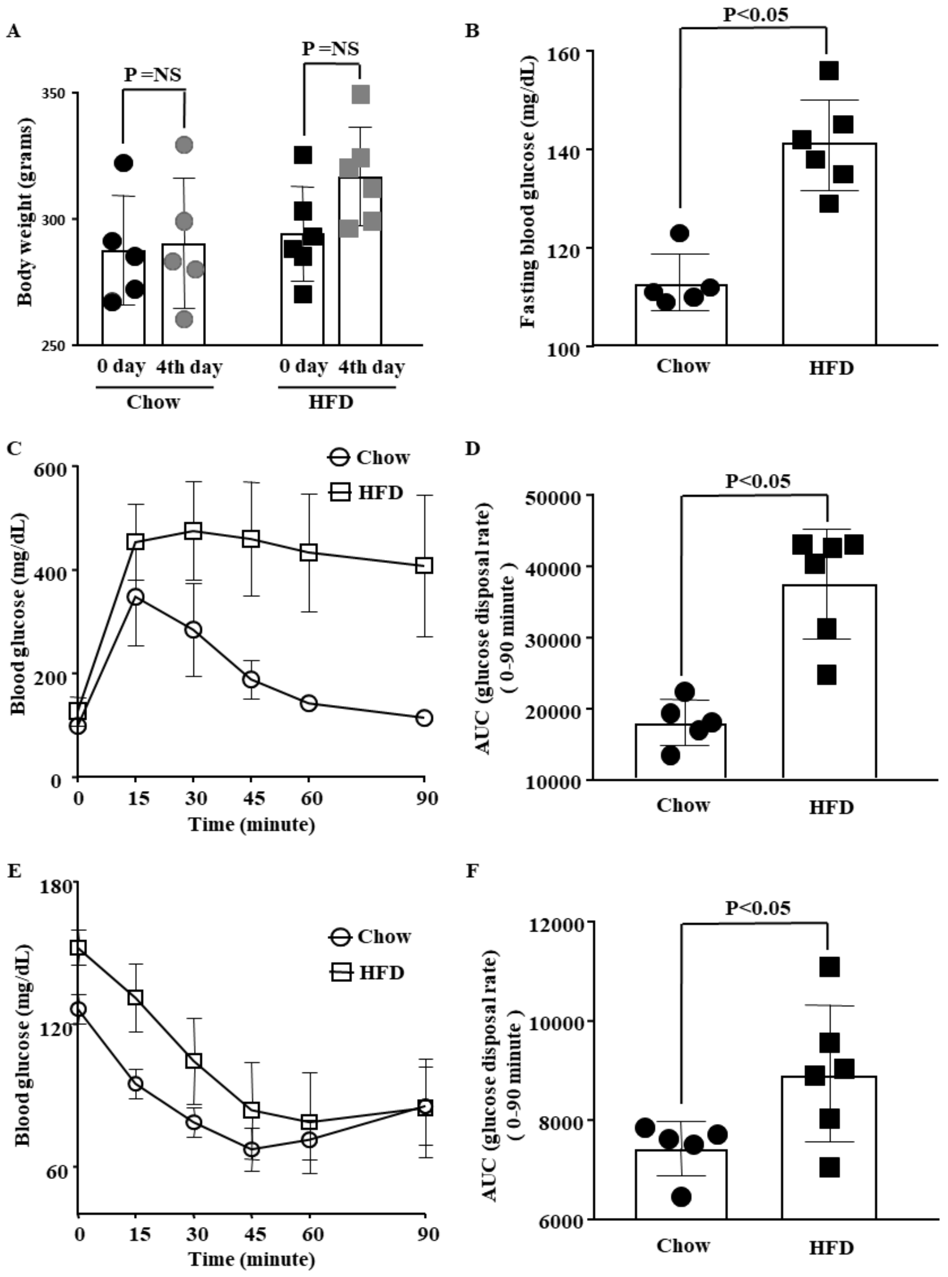

Supplementary figure 3.

High circulating FN-EDA in one-week HFD fed IR mice. (A) Bar diagram represents the circulating level of FN-EDA in female mice blood plasma following HFD feeding for one-week. HFD feeding for one-week significant increase FN-EDA in female mice. (B) Bar diagram depicts FPG in one-week HFD fed female mice. HFD feeding for one-week significant increases FPG in female mice compared to chow-fed mice. Line and bar graph represent glucose disposal following (C and D) IPGTT and (E and F) IPITT in female mice kept on HFD for one-week. Glucose disposal was significantly impaired overtime in HFD fed female mice following glucose and insulin tolerance tests. Data represented as mean (SD) and P<0.05 considered statistically significant.

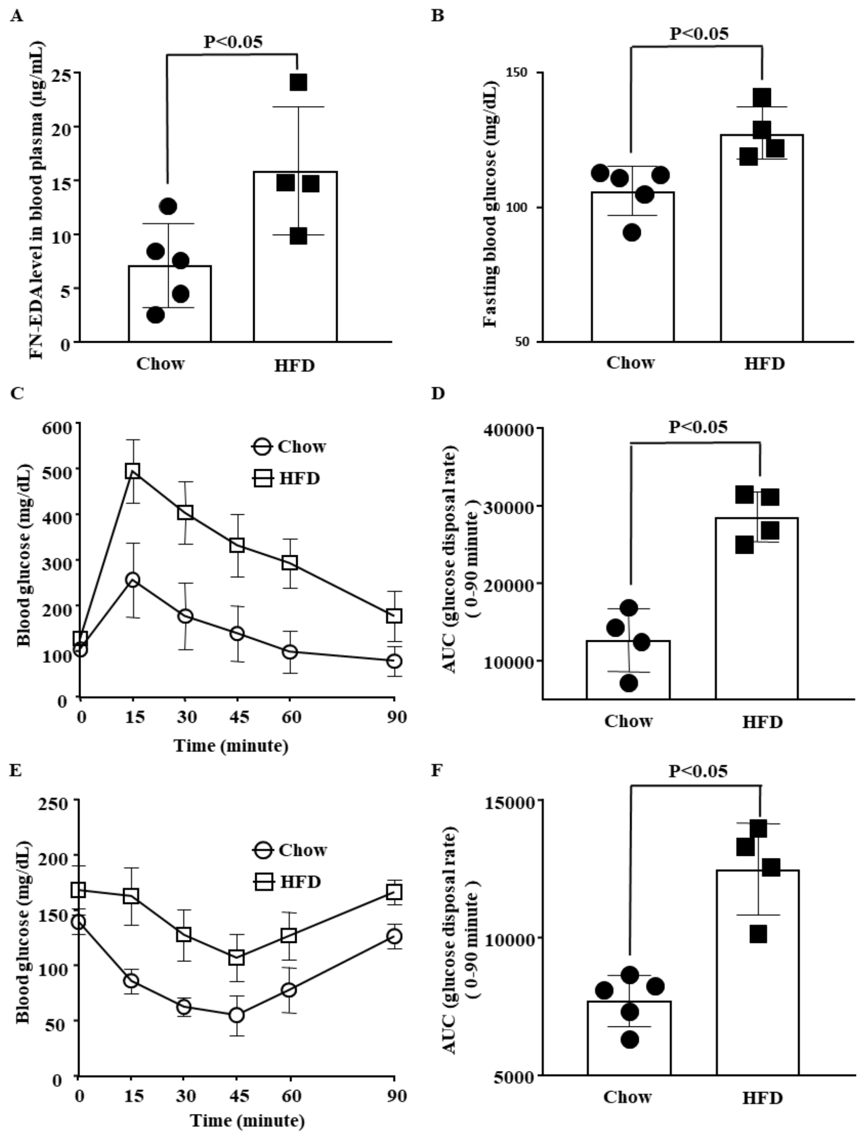

Supplementary figure 4.

TLR4 inhibitor protects from IR. (A) Mice on HFD for one-week were treated with a TLR4 inhibitor three times at 48 hours interval. The first dose was administered just before keeping the mice on HFD. The bar graph depicts AUC of glucose disposal following (B) IPGTT (C) IPITT in mice treated with TLR4 inhibitor (TAK-242, 1mg/kg). TLR4 inhibition significantly increased glucose utilization in HFD fed mice following glucose and insulin load. Data represented as mean (SD) and P<0.05 considered statistically significant.

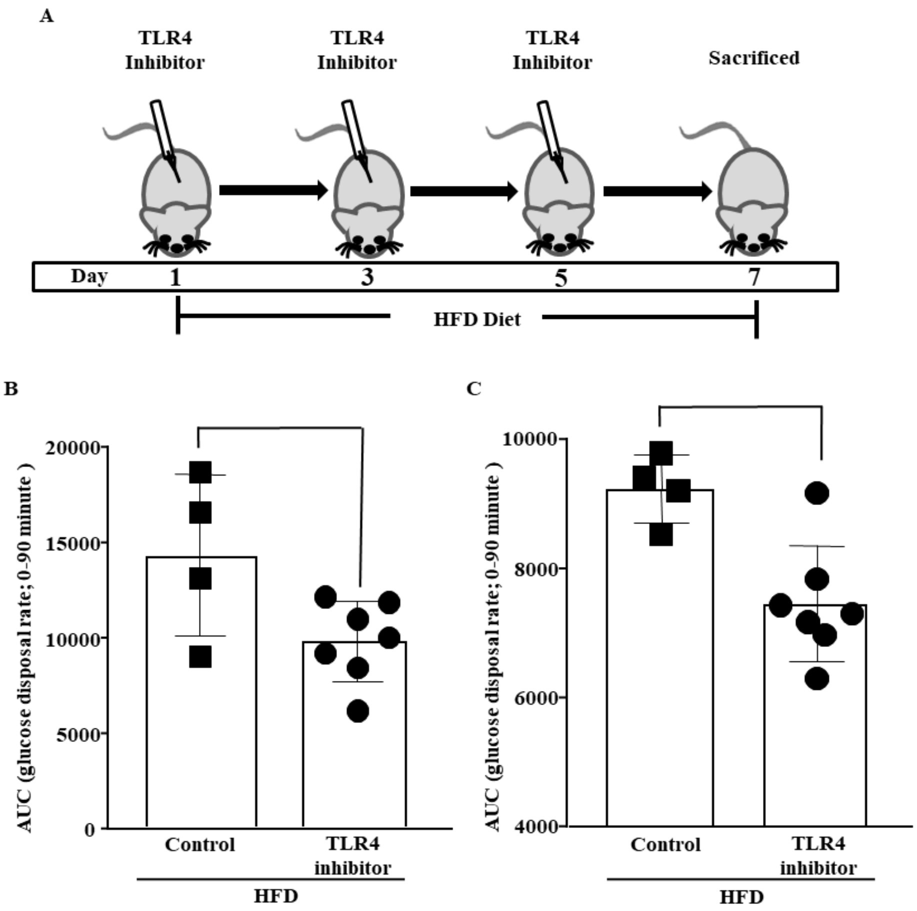

Supplement: Supplementary file 1 — Supplementary information. [file 41598_2020_65970_MOESM1_ESM.pdf]
